# Regulatory networks and 5′ partner usage of miRNA host gene fusions in breast cancer

**DOI:** 10.1002/ijc.33972

**Published:** 2022-02-26

**Authors:** Völundur Hafstað, Rolf Søkilde, Jari Häkkinen, Malin Larsson, Johan Vallon‐Christersson, Carlos Rovira, Helena Persson

**Affiliations:** ^1^ Department of Clinical Sciences Lund, Division of Oncology Lund University Cancer Centre, Faculty of Medicine Lund Sweden; ^2^ Department of Physics, Chemistry and Biology, National Bioinformatics Infrastructure Sweden, Science for Life Laboratory Linköping University Linköping Sweden

**Keywords:** breast cancer, fusion gene, fusion transcript, microRNA

## Abstract

Genomic rearrangements in cancer cells can create gene fusions where the juxtaposition of two different genes leads to the production of chimeric proteins or altered gene expression through promoter‐swapping. We have previously shown that fusion transcripts involving microRNA (miRNA) host genes contribute to deregulation of miRNA expression regardless of the protein‐coding potential of these transcripts. Many different genes can also be used as 5′ partners by a miRNA host gene in what we named recurrent miRNA‐convergent fusions. Here, we have explored the properties of 5′ partners in fusion transcripts that involve miRNA hosts in breast tumours from The Cancer Genome Atlas (TCGA). We hypothesised that firstly, 5′ partner genes should belong to pathways and transcriptional programmes that reflect the tumour phenotype and secondly, there should be a selection for fusion events that shape miRNA expression to benefit the tumour cell through the known hallmarks of cancer. We found that the set of 5′ partners in miRNA host fusions is non‐random, with overrepresentation of highly expressed genes in pathways active in cancer including epithelial‐to‐mesenchymal transition, translational regulation and oestrogen signalling. Furthermore, many miRNAs were upregulated in samples with host gene fusions, including established oncogenic miRNAs such as *mir‐21* and the *mir‐106b~mir‐93~mir‐25* cluster. To the list of mechanisms for deregulation of miRNA expression, we have added fusion transcripts that change the promoter region. We propose that this adds material for genetic selection and tumour evolution in cancer cells and that miRNA host fusions can act as tumour ‘drivers’.

## Graphical abstract

What's new?

Fusion transcripts have been identified in many tumour types, but it remains unclear how many of them represent functional tumour drivers versus passenger events. Among several mechanisms causing deregulation of miRNA expression in cancer, genomic rearrangements can create microRNA host gene fusions that alter microRNA expression regardless of the protein‐coding potential of the fusion transcript. The authors show that the 5′ partners of host fusions are highly‐expressed genes in subtype‐specific pathways active in breast cancer. Host fusions can thereby provide material for genetic selection and tumour evolution in cancer cells, with miRNA host fusions potentially acting as tumour drivers.

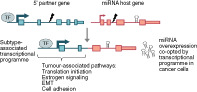

**Abbreviations**
CPM: counts per million reads
EMT: epithelial‐mesenchymal transition
ER: oestrogen receptor
FPKM: fragments per kilobase of exon model and million reads
GO: gene ontology
HER2: human epidermal growth factor receptor 2
IHC: immunohistochemistry
Kb: kilobase pairs
KEGG: Kyoto encyclopaedia of genes and genomes
Mb: megabase pairs
miRNA: microRNA
mRNA: messenger RNA
NMD: nonsense‐mediated mRNA decay
RNA‐Seq: RNA sequencing
RPPA: reverse phase protein array
SCAN‐B: Sweden Cancerome Analysis Network—Breast
snoRNA: small nucleolar RNA
TCGA: The Cancer Genome Atlas
WGS: whole genome sequencing

## 1 INTRODUCTION

The genomes of cancer cells can contain an extensive number of genetic alterations, ranging from point mutations affecting a single base pair to structural variants including copy‐number changes and translocations of chromosome segments. Epigenetic changes in DNA methylation and histone modifications can change chromatin states and further contribute to the deregulation of the genome.^1^ The relative insensitivity to the control systems and safeguards of normal cells leads to a remarkable flexibility in moulding the cancer genome to ultimately increase cell survival and proliferation. Genomic rearrangements can lead to juxtaposition of sequences from two different genes to create a fusion gene, sometimes resulting in a chimeric protein with altered properties, or in promoter‐swapping where the expression of one gene is placed under the control of the regulatory elements of the other gene.^2^ There are many well‐established examples of oncogenic gene fusions, some of which have been successfully exploited as targets for therapy.^3^, ^4^ Modern sequencing technology has greatly facilitated the detection of gene fusions and fusion transcripts have been identified in many tumour types.^5^ It is, however, still an open question how many of the detected fusion transcripts represent functional tumour drivers vs passenger events.

We have previously shown that the host genes of intronically encoded small noncoding RNAs including both microRNA (miRNA) and small nucleolar RNA (snoRNA) are overrepresented in fusion transcripts in breast cancer.^6^ Analyses of fusion transcripts have mainly focused on the production of chimeric proteins, but the co‐transcriptional processing of miRNAs from primary transcripts^7^ implies that the coding potential of the transcript is irrelevant from the perspective of miRNA expression. We coined the term miRNA‐convergent fusions to describe a class of fusion transcripts where the exact identity and function of the 5′ partner gene is unimportant, and in which multiple and different 5′ partners can drive the expression of a given miRNA.^6^ For these fusion transcripts, recurrence is therefore only defined as multiple occurrences of fusions involving the same miRNA host gene as a fusion partner.

The role of miRNAs and their associated Argonaute proteins in regulation of gene expression is well established, and primarily occurs through base‐pairing of the miRNA to partially complementary target sites in the mRNAs of target genes, leading to mRNA destabilisation or translational inhibition.^8^ A considerable number of miRNAs have been reported to act as tumour suppressors or oncogenes^9^ and several different mechanisms have been described for deregulation of miRNA expression in cancer.^10^ Examples include genomic copy number alterations, epigenetic factors such as promoter methylation status, post‐transcriptional regulation of miRNA processing—and gene fusions involving miRNA host genes have now also been added to this list. Our earlier work demonstrated that the 5′ partners of fusion transcripts involving a miRNA host gene as 3′ partner had higher expression than the 5′ partners of non‐host genes, and that specific miRNAs were upregulated in samples with host gene fusions.^6^

Here we have explored the properties of miRNA host gene fusions in breast tumours from the TCGA^11^ and SCAN‐B^12^ cohorts with the hypotheses that (a) the 5′ partner genes should belong to pathways and transcriptional programmes that reflect the tumour phenotype and (b) that there should be a selection for fusion events that shape miRNA expression to benefit the tumour cell through known hallmarks of cancer such as increased survival, proliferation, angiogenesis, or migration. We find that the 5′ partners of miRNA host genes are associated with higher expression and lower promoter methylation. They are regulated by key transcription factors in cancer cells and act in pathways related to the malignant phenotype. Finally, we identify fusion transcripts as mechanisms for upregulation of oncogenic miRNAs including *mir‐21* and the *mir‐106b~mir‐93~mir‐25* cluster in breast cancer.

## 2 MATERIALS AND METHODS

### 2.1 Fusion transcript prediction

FusionCatcher^13^ version 1.00 was used to extract fusion events from all available RNA‐sequencing (RNA‐Seq) data in the TCGA‐BRCA project. Fusions with the following flags were filtered from the analysis due to their high likelihood of being false positives: 1000genomes, 1K<gap<10K, adjacent, ambiguous, duplicates, ensembl_partially_overlapping, gap<1K, gencode_fully_overlapping, gencode_partially_overlapping, gencode_same_strand_overlapping, healthy, m0, multi, non_cancer_tissues, non_tumor_cells, refseq_partially_overlapping, tcga‐normal, ucsc_partially_overlapping, banned, bodymap2, cacg, conjoing, cta_gene, ctb_gene, ctc_gene, ctd_gene, distance1000bp, ensembl_fully_overlapping, ensembl_same_strand_overlapping, gtex, hpa, mt, pair_pseudo_genes, paralogs, readthrough, refseq_fully_overlapping, refseq_same_strand_overlapping, rp_gene, rp11_gene, rrna, similar_reads, similar_symbols, ucsc_fully_overlapping, ucsc_same_strand_overlapping. Coordinates from miRBase^14^ release 22 were used to map intronic miRNAs to host genes and fusions based on GENCODE release 27 gene identifiers.

### 2.2 Classification of samples

To reduce the number of TCGA samples with missing data, tumour ER and HER2 status were defined by the RNA expression of *ESR1* and *ERBB2*, respectively. For each receptor, the distribution of expression values in fragments per kilobase of exon model and million reads (FPKM) were compared for each immunohistochemically determined (IHC) status and defined an expression threshold value between positive and negative (Figure S1). The threshold for ER‐positive samples was ESR1 FPKM = 5.7 and for HER2‐positive samples ERBB2 FPKM = 73.5. IHC receptor status was available for all SCAN‐B samples and therefore used instead of an FPKM threshold. PAM50 molecular subtypes in the SCAN‐B cohort were obtained as previously described.^15^

### 2.3 Expression and promoter methylation analysis

Methylation and gene expression matrices were obtained from TCGA. For expression analysis, we calculated the average expression of each 5′ fusion partner for each category of 3′ partner gene (3′ host including/excluding miRNA and 3′ not host), as well as average expression in samples where the 5′ partner was not involved in fusion events. The equivalent analysis was performed for 3′ partner genes. For the promoter methylation analysis, we calculated the average methylation levels of CpG islands located within −1000 to +200 bases of the transcription start site for each gene. Student's *t*‐test was used to test for differences in log_2_‐transformed methylation beta values and expression levels between different groups of 5′ fusion partner genes.

### 2.4 Enrichment analyses

Gene sets were obtained from the Molecular Signatures Database version 7.2^16^ (MSigDB) using the MSigDBR package. The clusterProfiler^17^ R package was used to detect overrepresented GO, HALLMARK, KEGG and REACTOME gene sets in the 5′ fusion partners. The UniBind^18^ robust set of TF‐DNA interactions was used to detect any overrepresented targets of transcription factors acting on the promoter region of 5′ fusion partners. Promoter regions were defined as −1000 to +200 bases from the transcription start site. For each tumour subtype, three random sets of unique 5′ fusion partners of non‐hosts were selected to match the number of 5′ partners of miRNA hosts to compare gene lists of equal lengths, as the number of non‐host fusions was substantially larger than the number of miRNA host fusions. The average enrichment value for the random sets was calculated to represent gene set overrepresentation for the non‐host 5′ fusion partners. All 5′ partners of canonical snoRNAs hosts (scaRNAs, C/D and H/ACA box snoRNAs) were excluded from the analysis. The universe used for the gene set overrepresentation analysis contained all expressed genes in the TCGA‐BRCA cohort, defined as genes with 95th percentile FPKM >1 across the entire cohort. The *P*‐values for overrepresented gene sets were calculated using the hypergeometric distribution. The equivalent analysis was performed for 3′ partner genes.

### 2.5 Differential miRNA expression analysis

Total expression per mature miRNA in miRBase release 22^14^ was calculated with a custom Perl script from the TCGA miRNA isoform quantification files using converted genomic coordinates. Differential expression analysis was performed using the exactTest implemented in edgeR.^19^

### 2.6 Analysis of whole genome sequencing data

GRCh37‐lite reference alignments for whole genome sequencing (WGS) data were downloaded from the GDC Legacy Archive (https://portal.gdc.cancer.gov/legacy-archive). WGS and RNA‐Seq file identifiers were connected to TCGA barcodes using the Bioconductor package TCGAbiolinks version 2.16.0^20^ and R version 4.0.2. GRCh37 gene coordinates for the 5′ partners of *VMP1* fusion transcripts were obtained from GENCODE release 27.^21^ Reads aligning to the 5′ partners and *VMP1* were extracted from the WGS BAM files and discordant read pairs where one read aligned in each of the fusion partner genes were extracted. We considered the presence of at least one such read pair to confirm a fusion event at the DNA level. This analysis was done in Python version 2.7.15 using the module pysam version 0.15.3.^22^

### 2.7 Analysis of miRNA target genes

Expression values in counts per million reads (CPM) for miRNAs that were differentially expressed between tumours with host gene fusions and tumours without host fusions were used together with the FPKM values of all targets of those miRNAs as predicted by TargetScan^23^ for correlation tests using Pearson's product moment correlation coefficient. Correlation tests were also performed for miRNA expression against protein levels obtained from TCGA reverse phase protein array data (RPPA).

### 2.8 Experimental validation of fusion transcripts

Fusion transcripts for validation were selected among SCAN‐B tumours with available RNA and small RNA sequencing data. Transcripts where the fusion junction overlapped repetitive elements annotated by RepeatMasker were excluded to allow design of specific primers. Total RNA was treated with DNase I (ThermoFisher Scientific) and 200 ng was used for cDNA synthesis with random hexamers in 10 μL reactions using RevertAid H Minus reverse transcriptase (ThermoFisher Scientific) according to the manufacturer's instructions. The cDNA was diluted 1:3 with H_2_O and 2 μL were used in 15 μL reactions for real‐time quantitative RT‐PCR with iTaq Universal SYBR Green Supermix (Bio‐Rad) according to the manufacturer's instructions. For miRNAs, cDNA synthesis from 100 ng DNase‐treated total RNA was performed as described.^24^ Dilution of cDNA and PCR was done as before. Primer sequences are available in Table S1.

### 2.9 Statistical analyses

All statistical analyses were performed in R version 3.6.3‐4.0.5. The Benjamini‐Hochberg procedure was used to adjust *P*‐values in multiple testing to control the false discovery rate for all statistical tests.

## 3 RESULTS

### 3.1 Fusion transcripts in the TCGA breast cancer cohort

To explore 5′ partners and regulatory networks in miRNA host gene fusions, we have based our analyses on RNA‐Seq data for 1092 breast tumours from TCGA (TCGA‐BRCA cohort). For some analyses we have also included data for the 1540 breast tumours in our previous study (SCAN‐B cohort).^6^ There are many software tools available to detect fusion transcripts from RNA‐Seq data. We chose to use FusionCatcher^13^ due to its high sensitivity and comparatively low false discovery rate.^25^ Clinical data for the TCGA samples are summarised in Table 1. Several samples had missing or ambiguous status for the two main prognostic and treatment‐predictive biomarkers in breast cancer: oestrogen receptor alpha (ER, gene symbol *ESR1*) and Erb‐B2 receptor tyrosine kinase 2 (HER2, gene symbol *ERBB2*). We therefore decided to define ER and HER2 status by the expression of *ESR1* and *ERBB2*, respectively, since expression levels of these genes are available for every sample in the cohort from the RNA‐Seq data. Following this definition, 75% of samples were ER‐positive and 15% HER2‐positive, a distribution that is in‐line with current literature^26^ (see Table 1 and Figure S1). After applying the gene expression‐based cut‐off, the receptor status changed for 6.7% and 11.7% of samples previously annotated as positive or negative for ER or HER2, respectively. PAM50 molecular subtype classification was obtained from TCGA.^11^

**TABLE 1:** Clinical characteristics and molecular subtypes of the TCGA‐BRCA cohort

Age at diagnosis	
<35	29 (2.7%)
35‐50	269 (24.6%)
50‐75	652 (59.7%)
>75	140 (12.8%)
NA	2 (0.2%)
Gender	
Female	1081 (99.0%)
Male	11 (1.0%)
Tumour stage	
I	180 (16.5%)
II	619 (56.7%)
III	249 (22.8%)
IV	20 (1.8%)
Other/NA	24 (2.2%)
PAM50 status	
Luminal A	565 (51.7%)
Luminal B	213 (19.5%)
HER2‐enriched	82 (7.5%)
Basal	191 (17.5%)
Normal‐like	41 (3.8%)
ER status by IHC	
Positive	798 (73.1%)
Negative	237 (21.7%)
Intermediate	2 (0.2%)
NA	47 (4.3%)
HER2 status by IHC	
Positive	163 (14.9%)
Negative	557 (51.0%)
Equivocal	178 (16.3%)
Intermediate	12 (1.1%)
NA	174 (15.9%)
ER status by FPKM	
Positive	819 (75.0%)
Negative	273 (25.0%)
HER2 status by FPKM	
Positive	158 (14.5%)
Negative	934 (85.5%)
ER/HER2 status by FPKM	
ER+, HER2+	103 (9.4%)
ER+, HER2−	716 (65.6%)
ER−, HER2+	55 (5.0%)
ER−, HER2−	218 (20.0%)

In total, we detected over 274 000 fusion transcript events in 1092 samples, an average of 251 fusions per sample (Tables S2 and S3). A total of 16 530 unique genes were involved as fusion partners and 6293 of those occurred in more than one sample. There were no significant differences in the number of fusion events by PAM50 subtype, receptor status or tumour stage (Fisher's exact test, *P* = .334, *P* = .154, and *P* = .874, respectively). Only 2.2% of all unique fusion gene pairs were found among 111 samples annotated as normal breast tissue by TCGA, indicating that most of the detected fusions are specific to cancer (Table S2). Among all fusion events in tumours, 16 262 (5.9%) had a miRNA host gene as 3′ partner with a breakpoint located upstream of the intronic miRNA (miRNA‐including fusions). The number of miRNA‐excluding host gene fusions with the miRNA falling outside of the fusion product was similar with 15 674 events (5.7%). In total, we detected 672 miRNA host genes with 734 unique miRNAs as 3′ partners in fusion transcripts.

### 3.2 microRNA host genes are overrepresented in fusion transcripts

**FIGURE 1:**
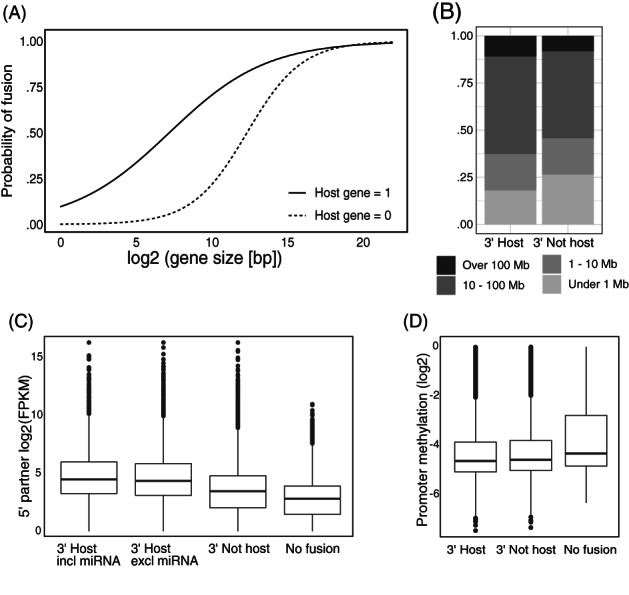
(A) MicroRNA hosts were significantly more likely than other genes to be detected in fusion transcripts. The plot shows logistic regression analysis with a model including host gene status, gene size and the interaction term. (B) Fusion partners in intra‐chromosomal fusion events were located further apart when the 3′ partner was a miRNA host vs a non‐host. (C) 5′ partners in miRNA host fusions had higher average expression than 5′ partners in non‐host fusions. The lowest expression of genes in the 5′ partner set was found in tumours where they were not involved in fusions. (D) Average promoter methylation levels for 5′ fusion partners were the lowest when the 3′ partner was a miRNA host, and lower for both host and non‐host fusions than in tumours where they were not involved in fusion transcripts

We have previously shown that miRNA host genes are overrepresented as fusion partners in the SCAN‐B breast cancer cohort.^6^ To confirm this in the TCGA data, we constructed a logistic regression model that considered miRNA host gene status, gene size, and the interaction between the two factors (Figure 1A). Host genes of miRNAs were indeed also overrepresented in the TCGA fusions (*P* = 7.62 × 10^−7^, Wald test). The model was limited to all expressed protein‐coding genes in the TCGA‐BRCA cohort.

Looking at the genomic location of fusion partners, 90.8% of fusion events were inter‐chromosomal and 9.2% were intra‐chromosomal. As shown in Figure 1B, the fusion partners of intra‐chromosomal fusions were significantly more likely to be further apart when the 3′ partner was a miRNA host (*P* < 2e − 16, Fisher's exact test). The median distance between fusion partners was 21.3 million base pairs (Mb) for miRNA hosts and 13.8 Mb for non‐hosts. No significant differences were observed in the distance between the two fusion partners when comparing miRNA‐including and miRNA‐excluding host gene fusions. Fusion transcripts linking closely located genes may be false positive events produced through readthrough transcription^13^; fusions flagged by FusionCatcher as joining adjacent genes or genes located less than 10 000 base pairs (Kb) apart were therefore removed before any analyses were performed.

### 3.3 5′ partners of miRNA host fusions are highly expressed and have lower promoter methylation

In accordance with the model of miRNA‐convergent fusions we hypothesise that there is a selection for 5′ partners that modulate miRNA expression in a way that provides an advantage to the cancer cell. We therefore analysed the properties of 5′ partners of miRNA host fusion transcripts, starting with their expression level. As seen in Figure 1C, the 5′ fusion partners of miRNA host genes had significantly higher expression levels than other 5′ partners (*P* < 2e − 16, Student's *t*‐test). The expression of 5′ fusion partners of miRNA hosts was also significantly higher for miRNA‐including fusions compared with miRNA‐excluding fusions (*P* = .01, Student's *t*‐test). Strikingly, 3′ fusion partners in fusions with miRNA hosts as 5′ partners instead had lower expression in miRNA‐including fusions (Figure S2, *P* = 5.00 × 10^−8^, Student's *t‐*test). Fusions with a miRNA host as 5′ partner preserve the host gene promoter and would not be predicted to change miRNA expression. Overall, genes had significantly higher expression in samples where they were detected in fusion events (*P* < 1e − 16), indicating that the probability of a gene taking part in a fusion is partly dependent on expression.

Since we observed that miRNA host genes have the most highly expressed 5′ fusion partners, we postulated that these 5′ partner genes may have more active promoter regions than other fusion partners. It is well established that DNA methylation levels influence promoter activity and accessibility for transcription factor binding. We therefore downloaded the corresponding TCGA methylation data and examined CpG islands in the region of −1000 to +200 bases around the transcription start site of each expressed protein‐coding gene. Average methylation levels across samples and CpG islands are shown in Figure 1D. Indeed, the 5′ fusion partners of miRNA hosts also had significantly lower methylation of promoter regions than other 5′ fusion partners (*P* = .01, Student's *t*‐test). In general, genes had lower promoter methylation in samples where they were involved in fusion transcripts (*P* < 2e − 16, Student's *t*‐test).

### 3.4 5′ fusion partners are enriched for genes involved in adhesion and the extracellular matrix

Next, we analysed the cellular pathway involvement of 5′ fusion partners to better understand the regulatory context of miRNA expression. We were interested both in comparing the 5′ partners of miRNA host vs non‐host fusion transcripts, as well as the 5′ fusion partner usage between different subgroups of breast tumours. We performed a gene set overrepresentation analysis and examined GO terms, KEGG pathways, HALLMARK and REACTOME gene sets obtained from the Molecular Signature Database (MSigDB).^16^ Only 5.9% of fusion transcripts had a miRNA in the 3′ partner gene, and therefore the total number of 5′ partners of miRNA hosts was much smaller than for non‐hosts (3525 vs 12 822 unique 5′ partner genes, respectively). This is problematic for the statistics of overrepresentation analysis, so we created gene lists of equal length for every tumour subgroup by randomly selecting equally large sets of non‐host 5′ partners. Host genes of canonical snoRNAs were removed from the lists of non‐host 5′ partners as they have also been shown to be overrepresented among fusion transcripts.^27^ As a background for the overrepresentation analyses we used all expressed genes in the TCGA‐BRCA cohort. All significant gene sets are included in Table S4.

**FIGURE 2:**
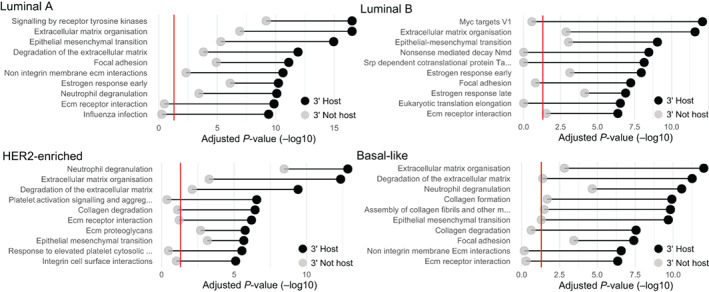
Gene set overrepresentation analysis of 5′ fusion partners of miRNA hosts vs non‐hosts, split by molecular subtype for the TCGA‐BRCA cohort. The plots show −log_10_ of the adjusted *P*‐values for the top 10 most significantly enriched KEGG, REACTOME, HALLMARK and GO pathways among 5′ partners of miRNA host fusions (black dots) together with the corresponding *P*‐values for non‐host fusions (grey dots) [Color figure can be viewed at wileyonlinelibrary.com]

Figure 2 shows the top 10 enriched gene sets among 5′ partners of miRNA host fusions divided by molecular subtype together with the corresponding *P*‐values for overrepresentation among 5′ partners of non‐host genes. Overrepresentation results for TCGA‐BRCA samples grouped by receptor status and for the SCAN‐B cohort are shown in Figure S3. Gene sets related to the extracellular matrix, focal adhesion and epithelial‐to‐mesenchymal transition (EMT) were consistently among the most overrepresented, regardless of molecular subtype or 3′ miRNA host status. This was also observed in the SCAN‐B data (Figure S3). Furthermore, oestrogen response genes were overrepresented among fusion partners for both miRNA host and non‐host fusions in the Luminal A and B subtypes, which mainly contain ER‐positive tumours. Interestingly, several gene sets related to translation, including eukaryotic translation initiation and elongation, SRP‐dependent co‐translational protein targeting to membrane, and nonsense‐mediated decay (NMD) were overrepresented regardless of subtype among the 5′ partners of miRNA host gene fusions, but not among non‐host fusions. Additional subtype‐specific gene sets with enrichment exclusively in miRNA host fusions included keratinisation in Basal‐like tumours (*P* = .0069, Fisher's exact test), as well as tight junctions and ‘pathways in cancer’ in Luminal A tumours (*P* = 5.01e − 6 and 3.66e − 8, respectively, Fisher's exact test).

We performed the analogous enrichment analysis for 3′ partner genes in fusions with miRNA hosts as 5′ partners for the different molecular subtypes in the TCGA data (Table S5). Since these fusions retain the promoter of the host gene they are not predicted to change miRNA expression. Gene sets related to the extracellular matrix, cell adhesion, and EMT were again significantly overrepresented with no clear difference between miRNA host and non‐host fusions. Interestingly, there was no difference between host and non‐host fusions for the gene sets related to protein translation that were overrepresented specifically among 5′ partners in 3′ miRNA host fusions. Furthermore, none of the aforementioned gene sets with subtype‐specific overrepresentation exclusively in 5′ partners of miRNA host fusions were significantly overrepresented in the 3′ partner analysis. These results indicate that the 5′ partners of miRNA host gene fusions have distinct functional characteristics.

### 3.5 Key transcription factors driving expression of the 5′ partners of miRNA host fusions

After concluding that the 5′ partners of fusion transcripts show strong enrichment for specific pathways, some clearly related to the tumour phenotype, we proceeded to analyse the underlying transcriptional networks. We based our analyses on the UniBind robust set of experimentally supported transcription factor‐DNA interactions^18^ and defined the promoter regions as −1000 to +200 bp around the transcription start site for each gene. Comparisons between miRNA host and non‐host fusions were performed by creating equally sized sets of random non‐host 5′ partners, as in the pathway overrepresentation analysis. All significant transcription factors are included in Table S4.

**FIGURE 3:**
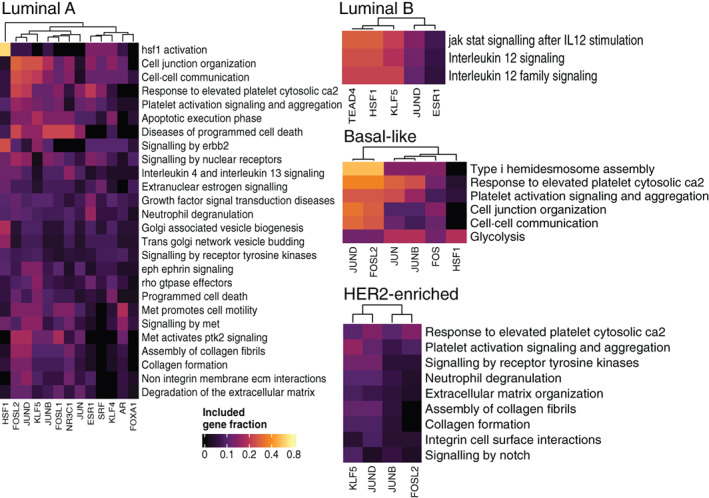
Enriched REACTOME pathways and transcription factor targets for the 5′ fusion partners of miRNA hosts, split by molecular subtype for the TCGA‐BRCA cohort. Colour intensity shows the fraction of genes in each REACTOME pathway that is regulated by a given transcription factor, as predicted by UniBind [Color figure can be viewed at wileyonlinelibrary.com]

Figure 3 shows enriched transcription factors and associated REACTOME pathways for the molecular subtypes, with the heatmaps indicating what fraction of the 5′ fusion partners in each pathway that are targets for a specific transcription factor. Results for tumours grouped by receptor status are shown in Figure S4. Target genes of the different paralogs of FOS and JUN, subunits of activator protein 1 (AP‐1), were overrepresented in all subtypes and in many cases among 5′ partners for both miRNA hosts and non‐host genes. Kruppel‐like factor 5 (KLF5) targets were enriched in the Luminal A and B, as well as HER2‐enriched subtypes, but not in Basal‐like tumours. We also observed an overrepresentation of ESR1 target genes in the predominantly ER‐positive Luminal A and B subtypes. The Luminal A subtype furthermore had specific enrichment among miRNA host fusions of targets for serum response factor (SRF), the glucocorticoid receptor (NR3C1) and forkhead box A1 (FOXA1), a modulator of ESR1 signalling. In the Luminal B subtype, target genes of both ESR1 and TEA Domain Transcription Factor 4 (TEAD4) were overrepresented specifically among the 5′ partners of miRNA host fusions. Strikingly, the transcription factors AP‐1, SRF, and TEAD4 have all been linked to epithelial‐to‐mesenchymal transition (EMT)^28^, ^29^, ^30^ and heat shock factor 1 (HSF1), enriched in both Luminal A and Basal‐like tumours, has been associated with many aspects of malignancy.^31^ These findings recapitulate the results of the cellular pathway analysis, highlighting the use of key regulatory networks in driving fusion transcript expression.

### 3.6 Upregulation of oncogenic miRNAs in fusion transcripts

Fusion transcripts that place miRNAs under the control of new promoters and pathways could lead to functional changes in the cancer cell. To study the effect of host gene fusions on miRNA expression we therefore performed a differential expression analysis of miRNAs between tumours where their host gene was a 3′ partner in a fusion and tumours without fusions involving the host gene.

**FIGURE 4:**
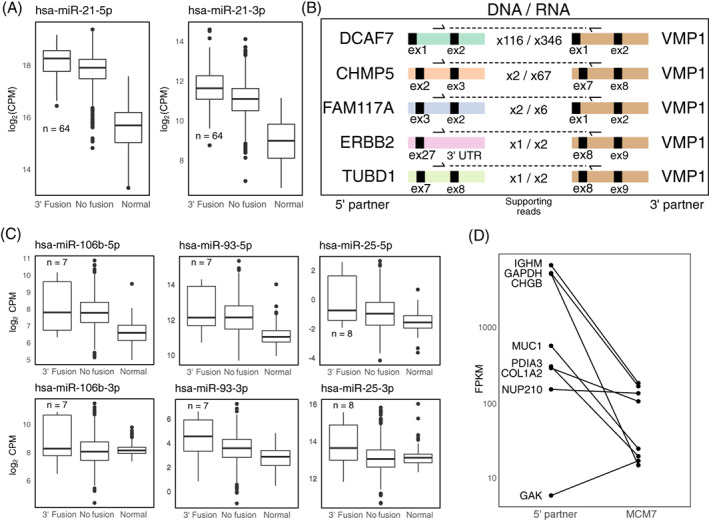
Expression levels of miR‐21‐5p and miR‐21‐3p were the highest in tumours where the host gene *VMP1* was a 3′ partner in a fusion transcript compared with tumours without *VMP1* fusions and normal tissue samples. (B) Validation of fusion events involving *VMP1* as a 3′ fusion partner including number of supporting reads in RNA‐Seq and DNA (WGS) data. In all cases *mir‐21* was part of the fusion transcript. (C) Expression levels for the mature miRNAs of the *mir‐106b~mir93~mir‐25* cluster in tumours with 3′ fusions of the host gene *MCM7* compared with tumours without *MCM7* fusions and normal tissue samples. (D) Individual expression levels for both fusion partners in samples with fusion transcripts involving *MCM7* as 3′ partner [Color figure can be viewed at wileyonlinelibrary.com]

A total of 80 unique miRNAs had significantly higher expression in tumours where their host gene was a 3′ partner in a fusion event compared with samples without fusions involving the host gene (Table S6). No miRNAs were underexpressed in their respective fusion samples, indicating that miRNA host fusions primarily are associated with upregulation of the included miRNA. The other chromosome copy may also still contain a functional host gene and miRNA. We and others have previously reported fusion transcripts involving the miRNA precursor *mir‐21* and its host gene *VMP1*,^6^, ^32^ and several papers have reported *VMP1* fusions without noting the presence of *mir‐21*.^33^, ^34^, ^35^, ^36^ Here we found fusions with *VMP1* as 3′ partner in 64 tumours (5.9% of the samples). There were 88 different fusion transcripts using 56 unique 5′ partner genes with the most common partners being *RPS6KB1* (10 fusions), *DCAF7* (5 fusions) and *ACTB* (4 fusions). For comparison, only 8 samples (0.7%) had 5′ fusions involving *VMP1*. As shown in Figure 4A, the mature miRNA products miR‐21‐5p and miR‐21‐3p were both significantly upregulated in tumours with 3′ *VMP1* fusions compared with tumours without host gene fusions (*P* = 2.2e − 7 and *P* = 1.2e − 22, edgeR exact test).^19^ Five samples with *VMP1* fusion transcripts had matched WGS data, all confirming the existence of these fusions at the DNA level (Figure 4B).

Several mature miRNAs of the *mir‐106b~mir‐93~mir‐25* cluster were also significantly overexpressed in tumours with 3′ fusions of the host gene *MCM7* (Figure 4C). The corresponding *P*‐values for miR‐106b‐5p and ‐3p were .0011 and 6.5e − 7, for miR‐93‐5p and ‐3p .013 and 2.7e − 7, and for miR‐25‐5p and ‐3p 9.8e − 4 and 1.6e − 5 (edgeR exact test).^19^ Fusions of MCM7 have previously been reported in the SCAN‐B breast cancer cohort,^6^ as well as in ovarian and prostate cancer from the TCGA project.^5^ Here we found fusion transcripts with *MCM7* as 3′ partner in eight tumours, however none of them had WGS data available. All fusions used different 5′ partners, most of them with higher expression than the host gene (Figure 4D).

To further analyse the impact of miRNA upregulation on tumour biology, we used the TCGA RNA‐Seq data to perform global correlation tests for the differentially expressed mature miRNAs of *mir‐21* and the *mir‐106b~mir‐93~mir‐25* cluster against all their putative target genes as predicted by TargetScan.^23^ We also calculated correlations between these miRNAs and the TCGA reverse phase protein array (RPPA) data (Table S7). For miR‐21‐5p there were 539 predicted target genes with significant negative correlation at the mRNA level, 105 of them with direct experimental evidence according to TarBase v8^37^ and 4 significantly negatively correlated proteins that were predicted targets (none of them in TarBase). The six mature miRNAs in the *mir‐106b~mir‐93~mir‐25* cluster together had 1390 significantly negatively correlated predicted target mRNAs, 502 with direct experimental evidence and 17 significantly negatively correlated predicted target proteins (9 in TarBase).

Many studies have reported oncogenic functions for miR‐21‐5p and the *mir‐106b~mir‐93~mir‐25* cluster, which is also paralogous to the *mir‐17~92* and *mir‐106a~mir‐363* clusters.^38^, ^39^, ^40^ For example, the tumour suppressor *PTEN* has been shown to be regulated by both miR‐21‐5p and miR‐106b‐5p.^41^, ^42^ We found that PTEN mRNA levels were significantly negatively correlated with both miR‐21‐5p, miR‐106b‐5p, and miR‐25‐3p (Pearson's *r* = −.23, −.26 and −.16; *P* = 2.4e − 10, 2.7e − 20 and 2.5e − 8, respectively). Other negatively correlated validated targets for miR‐21‐5p included the mRNAs of *LIFR*, a metastasis suppressor in breast cancer,^43^ and *SPRY2*, a negative regulator of RAS and MAPK signalling,^44^ with Pearson correlations of −0.46 and −0.42, respectively (*P* = 4.9e − 60 and 5.4e − 48). Validated targets for miR‐106b‐5p with negative correlation at the mRNA level included the tumour suppressors *RB1* and *RBL2* ^45^ (Pearson's *r* = −.32 and −.27; *P* = 3.4e − 26 and 5.9e − 17).

From these data it is also possible to identify putative new target genes that have been predicted as targets by TargetScan and that are negatively correlated with the miRNAs across the TCGA‐BRCA samples. Examples for miR‐21‐5p are the proteins of SRSF1, a regulator of alternative splicing,^46^ and XBP1, a transcription factor involved in the unfolded protein response^47^ (Pearson's *r* = −.27 and −.26; *P* = 8.8e − 9 and 3.4e − 8). *ESR1*, the oestrogen receptor alpha, was predicted as a target for miR‐106b‐5p and miR‐93‐3p, and both miRNAs were negatively correlated with the mRNA (Pearson's *r* = −.20 and −.15; *P* = 1.3e − 7 and .02). ESR1 protein was also negatively correlated with miR‐106b‐p using two different antibodies (Pearson's *r* = −.36 and −.18; *P* = 3.1e − 31 and 3.0e − 5). Furthermore, *PEA15*, a multifunctional protein involved in DNA damage response,^48^ was predicted as a target for miR‐106b‐5p and miR‐25‐5p and was negatively correlated on the protein, but not mRNA, level (Pearson's *r* = −.22 and −.15; *P* = 3.9e − 9 and .006).

Finally, we also selected 11 tumours with miRNA host gene fusions and available RNA from the SCAN‐B breast cancer cohort for experimental validation by real‐time quantitative RT‐PCR. Seven out of the 11 samples (64%) had a readily detectable fusion transcript and for 9 out of the 11 samples (82%) the miRNA was expressed above the median level of a control group of six tumours (Table S8 and Figure S5).

## 4 DISCUSSION

Here we have investigated the 5′ partners that drive expression of fusion transcripts involving miRNA host genes in breast tumours from the TCGA and SCAN‐B cohorts. MicroRNA host genes are enriched among fusion partners in both datasets and we hypothesised that the 5′ partners would be part of cellular pathways and transcriptional programmes that were relevant to the tumour phenotype. We found that the 5′ partner genes used by miRNA hosts as a set have higher expression and lower promoter methylation than the 5′ partners of non‐host fusion transcripts. The 5′ partners of both host and non‐host fusions were strongly enriched for genes related to the extracellular matrix, focal adhesions and EMT in all tumour subgroups, and oestrogen response genes were enriched in the Luminal A and B subtypes that are dominated by ER‐positive tumours. A few pathways were specifically enriched for partners of miRNA host genes including translation and NMD in all molecular subtypes, keratinisation in basal‐like tumours and tight junctions in the Luminal A subtype.

**FIGURE 5:**
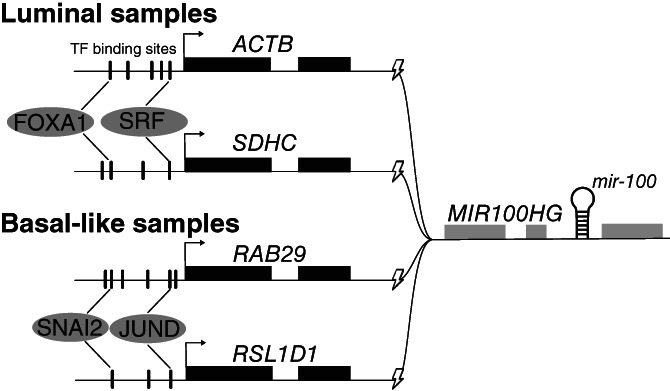
This schematic figure of fusion transcripts involving *mir‐100* illustrates how the expression of a 3′ fusion partner and its intronic miRNA can be regulated by the same transcription factors across many fusion events, even when the 5′ fusion partner genes are different. We have observed that 5′ partners of miRNA host genes tend to be regulated by transcription factors that are of biological relevance to the samples' molecular subtype

The creation of fusion transcripts provides a mechanism for uncoupling the expression of specific miRNAs from their host genes and normal transcriptional programme, and the transcription factors that were overrepresented in the promoters of 5′ partner genes function in different aspects of malignancy. The transcription factors AP‐1, SRF and TEAD4 have all been linked to EMT,^28^, ^29^ reflecting the enrichment of gene sets related to the extracellular matrix and cell adhesion. In cancer cells, HSF1 drives a complex transcriptional programme including, for example, regulation of the cell cycle, metabolism, adhesion and translation^31^ and both KLF4 and KLF5 regulate proliferation and apoptosis, although potentially with opposing effects.^49^ Overrepresentation of targets for ESR1 and FOXA1 in the luminal subtypes is in line with the cooperation between these two transcription factors and the importance of oestrogen signalling in these tumours.^50^ Figure 5 shows an example of how key transcription factors can deregulate miRNA expression through convergent fusions using different 5′ partners.

We furthermore hypothesised that the fusion transcripts should change miRNA expression to benefit the tumour cell through regulation of the known hallmarks of cancer such as increased survival, proliferation, angiogenesis or migration. Differential expression analysis identified a number of miRNAs that were upregulated in tumours with fusions involving their host gene. These included the well‐established oncogenic miRNAs *mir‐21* in *VMP1* and the *mir‐106b~mir‐93~mir‐25* cluster in *MCM7*. Expression of these miRNAs was anticorrelated with many predicted and experimentally validated target genes across the TCGA breast cancer cohort. These included many tumour suppressors, which is in line with the proposed oncogenic role for the miRNAs,^41^ but there were also many oncogenes among the negatively correlated genes (Table S7). This suggests that the functions of these miRNAs might be more complex than what has previously been appreciated, and that there might be subgroups of tumours where their roles are different. Since cellular signalling pathways normally contain both positive and negative regulators, this is not necessarily an unexpected finding, but a clear reminder that no miRNA evolved for the purposes of a cancer cell.

Although several papers have identified fusion transcripts in cancer,^5^ global analyses of miRNA host gene fusions are still lacking with the exception of our work in breast cancer.^6^ We have previously reported that fusions involving miRNA hosts are common but overlooked for several reasons; research has focused on protein‐coding genes and in‐frame fusion transcripts, and miRNA‐convergent fusions using different 5′ partners may not have been classified as recurrent. One advantage with the TCGA data is that more than 98% of the samples have small RNA sequencing data, which allowed us to analyse the effects of host fusions on miRNA expression. Still, the number of samples with fusions for a given host gene becomes limiting for the ability to detect differentially expressed miRNAs. Whole genome sequencing data was also available from TCGA for 5 out of the 64 tumours with *mir‐21* fusions, allowing us to verify the genomic fusions involving *mir‐21*.

The expression of miRNAs can be deregulated in many different ways, including genomic amplification and deletion, altered promoter methylation, transcriptional rate, or processing. To this list we have added convergent fusion transcripts, potentially caused by genomic rearrangements that change the promoter region. We have shown that the set of 5′ partners in miRNA host fusions is non‐random and demonstrates overrepresentation of highly expressed genes in subtype‐specific pathways active in breast cancer. This adds material to the process of genetic selection and tumour evolution in cancer cells and suggests that miRNA host fusions may provide a growth advantage and function as tumour ‘drivers’.

## CONFLICT OF INTEREST

The authors do not declare any conflicts of interest.

## ETHICS STATEMENT

For the SCAN‐B data the study was conducted in accordance with the Declaration of Helsinki and has been approved by the Regional Ethical Review Board of Lund (2007/155, 2009/658, 2009/659, 2014/8), the county governmental biobank centre, and the Swedish Data Inspection group (364‐2010). Written information was given by trained health professionals and all patients provided written informed consent.

## Supporting information


**Appendix S1** Supporting Information.

**Table S2** Fusion transcripts detected by FusionCatcher in the TCGA BRCA cohort. Information about the intronic miRNA is given if the 3′ fusion partner is a miRNA host. miRNAs located outside the transcript are labelled as ‘MIREXCL’. Fusion pairs that also were detected in samples annotated as normal breast tissue by TCGA are marked ‘1’.

**Table S3** Fusion transcript classification according to FusionCatcher for all fusion transcripts and transcripts including a miRNA within the 5′ or 3′ fusion partner, respectively. The table includes all fusion transcripts that pass filtering criteria, also when there are multiple transcripts involving the same partner genes in a single sample.

**Table S4** Results of gene set overrepresentation analysis of 5′ fusion partners for the TCGA and SCAN‐B datasets. Overrepresentation was calculated using the clusterProfiler R package. For each dataset, the fusions were split both by tumour ER/HER2 status and by molecular subtype. GO, KEGG, HALLMARK and REACTOME gene sets from MSigDB were analysed. The tables also contain transcription factor target gene enrichment obtained from UniBind. For each dataset and subtype analysed, a *P*‐value was calculated for 5′ partners of miRNA hosts (3′ host *P*‐adj) and for non‐hosts (3′ non‐host *P*‐adj). For the non‐hosts, a random subsampling of 5′ partners was performed to analyse a gene list of the same length as for the corresponding miRNA host 5′ partners. The sampling was done in triplicate and the mean adjusted *P*‐value is displayed in the 3′ non‐host *P*‐adj column.

**Table S5** Results of gene set overrepresentation analysis of 3′ fusion partners for the TCGA dataset. Overrepresentation was calculated using the ClusterProfiler R package. GO, KEGG, HALLMARK and REACTOME gene sets from MSigDB were analysed for each molecular subtype. The table also contains transcription factor target gene enrichment obtained from UniBind. For each subtype analysed, a *P*‐value was calculated for 3′ partners of miRNA hosts (5′ host *P*‐adj) and for non‐hosts (5′ non‐host *P*‐adj). For the non‐hosts, a random subsampling of 3′ partners was performed to analyse a gene list of the same length as for the corresponding miRNA host 3′ partners. The sampling was done in triplicate and the mean adjusted *P*‐value is displayed in the 3′ non‐host *P*‐adj column.

**Table S6** Correlation tests in TCGA data for the miRNAs shown in Figure 4. The correlation tests were done on the expression levels of miRNAs and their putative targets as predicted by TargetScan, and between miRNAs and protein levels as reported by protein expression arrays. Only significant correlations are reported. Information about miRNA target prediction is given in the form of cumulative context++ scores and number and types of binding sites on the target mRNA. Site types as explained by TargetScan: 8mer: An exact match to positions 2 to 8 of the mature miRNA (the seed + position 8) followed by an ‘A’. 7mer‐1A: An exact match to positions 2 to 7 of the mature miRNA (the seed) followed by an ‘A’. 7mer‐m8: An exact match to positions 2 to 8 of the mature miRNA (the seed + position 8). 6mer: An exact match to positions 2 to 7 of the mature miRNA (the seed). Non‐canonical: Extensive complementarity outside the seed region.

**Table S7** Correlation tests in TCGA data for the miRNAs shown in Figure 4. The correlation tests were done on the expression levels of miRNAs and their putative targets as predicted by TargetScan, and between miRNAs and protein levels as reported by protein expression arrays. Only significant correlations are reported. Information about miRNA target prediction is given in the form of cumulative context++ scores and number and types of binding sites on the target mRNA. Site types as explained by TargetScan: 8mer: An exact match to positions 2 to 8 of the mature miRNA (the seed + position 8) followed by an ‘A’. 7mer‐1A: An exact match to positions 2 to 7 of the mature miRNA (the seed) followed by an ‘A’. 7mer‐m8: An exact match to positions 2 to 8 of the mature miRNA (the seed + position 8). 6mer: An exact match to positions 2 to 7 of the mature miRNA (the seed). Non‐canonical: Extensive complementarity outside the seed region.

## Data Availability

The results published here are in part based upon data generated by The Cancer Genome Atlas managed by the NCI and NHGRI. Information about TCGA can be found at http://cancergenome.nih.gov. dbGAP accession number phs000178.v11.p8. Due to Swedish law, the patient consent, and the risk that the sequence data contain person‐identifiable information and hereditary mutations, we cannot deposit the SCAN‐B raw sequence data in a repository. The dataset supporting the conclusions of this article is available in the NCBI Gene Expression Omnibus (GEO) repository, accession number GSE96058, and from the corresponding author upon request.
