# Supplementary material for: Regulatory networks and 5′ partner usage of miRNA host gene fusions in breast cancer
Source: Int J Cancer. 2022 Feb 26;151(1):95–106. doi: 10.1002/ijc.33972 (PMC9303785; doi:10.1002/ijc.33972)
Supplement: Supplementary file 1 — Appendix S1 Supporting Information. [file IJC-151-95-s007.pdf]

Regulatory networks and 5' partner usage of miRNA host gene fusions in breast cancer

Völundur Hafstað. Rolf Søkilde. Jari Häkkinen. Malin Larsson. Johan Vallon-Christersson, Carlos Rovira & Helena Persson

Table of Contents

Supplementary Figure S1……………………………………………………………….....2
Supplementary Figure S2……………………………………………………………….....3
Supplementary Figure S3……………………………………………………………….....4
Supplementary Figure S4……………………………………………………………….....5
Supplementary Figure S5……………………………………………………………….....6
Supplementary Table S1………………………………………………………………….7
Supplementary Table S8………………………………………………………………….9

Supplementary Tables S2-S7 are available separately as .xlsx files.

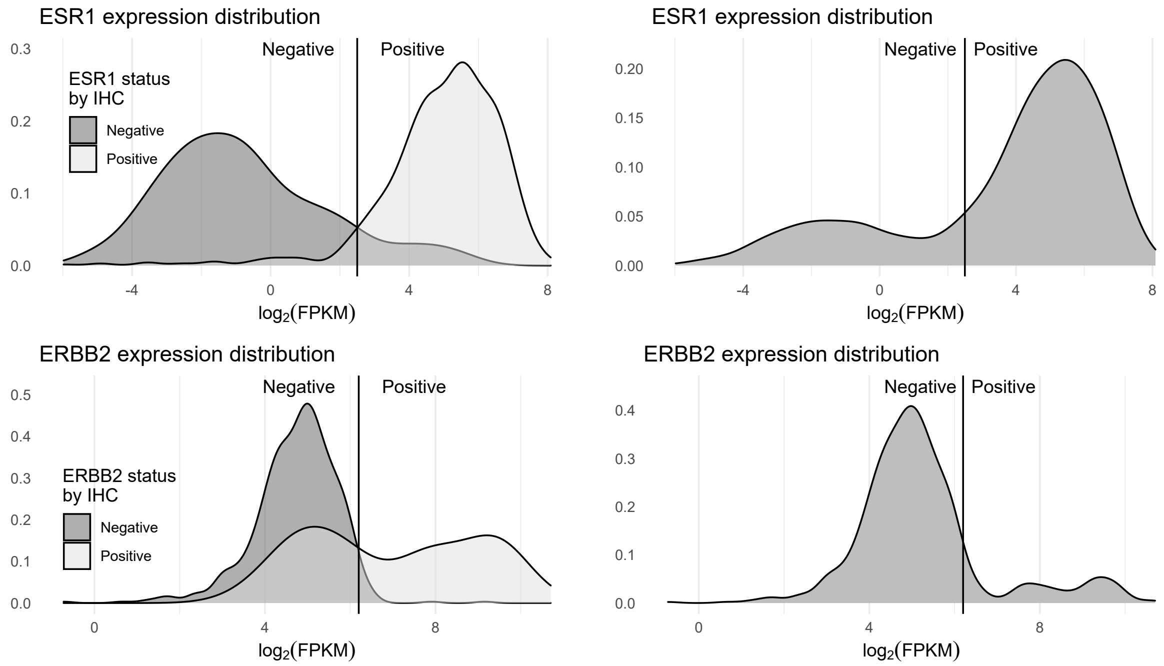

Supplementary Figure S1. Expression distribution of ESR1 and ERBB2 by IHC status and globally in the TCGA-BRCA cohort. Shown is the FPKM threshold values used to define ER- and HER2-positive/negative samples as determined by IHC distribution.

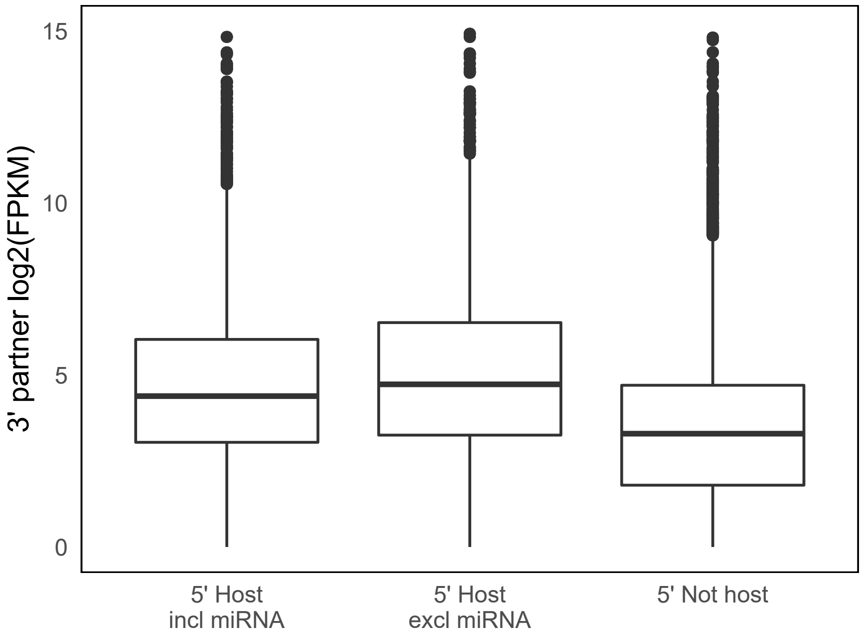

Supplementary Figure S2. Fusion transcripts with miRNA hosts as 5' partner preserve the promoter of the host gene and are not predicted to change miRNA expression. They differ from 3' miRNA host fusions in having partners with lower expression in fusion transcripts that include the position of the miRNA than in miRNA-excluding host gene fusions. Irrespective of whether the miRNA is included. their partners still have higher average expression than non-host fusions.

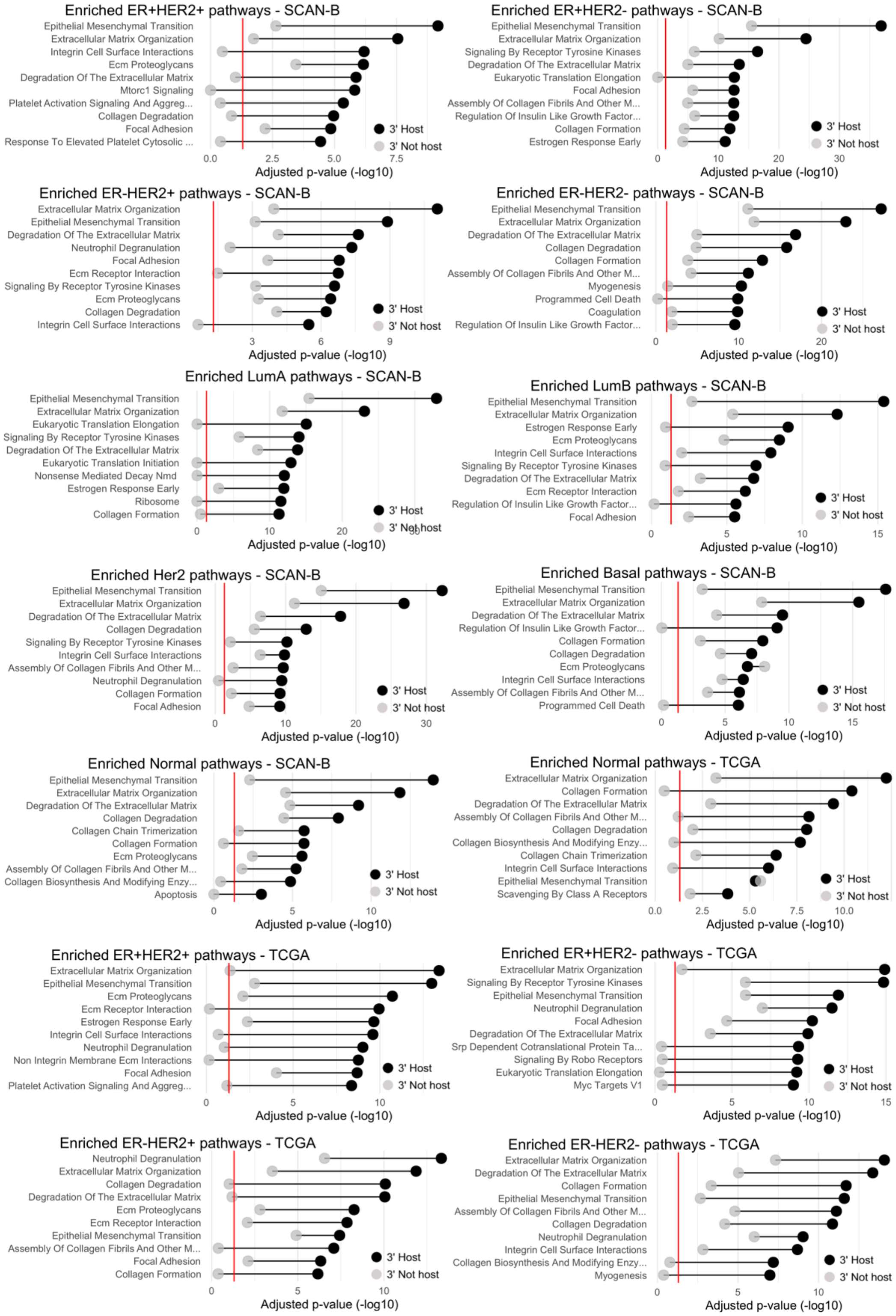

Supplementary Figure S3. Gene overrepresentation analysis of 5' fusion partners of miRNA hosts *vs* non-hosts in the TCGA-BRCA and SCAN-B cohorts. Samples are split by receptor status and molecular subtypes for the SCAN-B cohort and by receptor status for the TCGA-BRCA cohort.

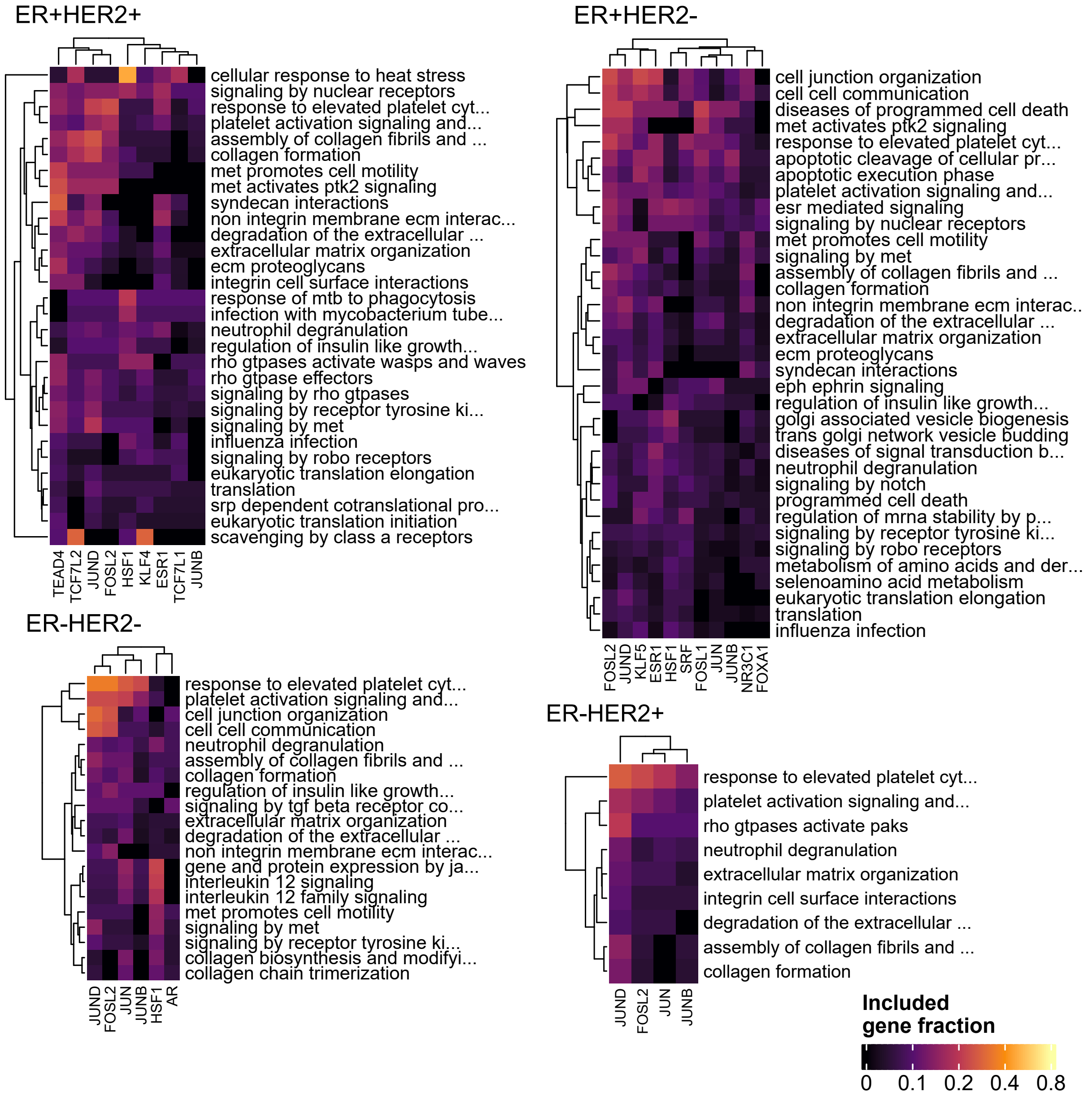

Supplementary Figure S4. Enriched REACTOME pathways and transcription factor targets in the 5' fusion partners of miRNA hosts. split by receptor status. Colour intensity shows the fraction of genes in each REACTOME pathway that are regulated by each transcription factor. as predicted by UniBind.

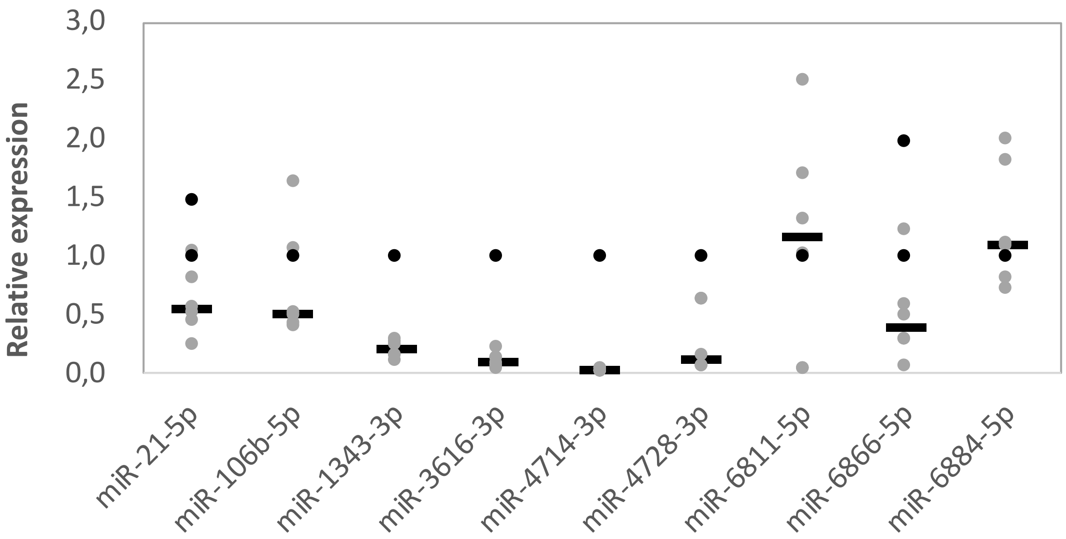

Supplementary Figure S5. Real-time quantitative RT-PCR measurement of miRNA expression for 11 tumours with host gene fusions selected from the SCAN-B cohort. Relative expression was calculated as $2^{-\Delta Cq}$. Filled. black circles represent samples with fusions and filled. grey circles six additional tumours randomly selected among the 11 samples. The horizontal bars mark the median for the six control tumours.

Supplementary Table S1. Primers used for real-time quantitative RT-PCR validation of fusion transcripts and measurement of miRNA expression. For each fusion an assay was designed with a forward primer in the 5' partner and a reverse primer in the 3' partner. Matched positive control assays were created where one of the primers was matched with another primer in the same gene (3' partner when possible). MRPL19 was used a positive control for all samples. SNORD48 was used as a positive control for small RNA PCRs.

5' partner	3' partner	Primer name	Sequence	Notes
B3GNTL1	VMP1	B3GNTL1_5FP	TGGTTGCAGAGTGAGGAGAG	
B3GNTL1	VMP1	VMP1_3RP	GTGCCCATTTCGCTTTTGTG	
B3GNTL1	VMP1	VMP1_3FP	ATAGTGGAGCAAATGGTGGC	Control assay
PITPNC1	VMP1	PITPNC1_5FP	GAGAACATGGCTGAGCTCAC	
PITPNC1	VMP1	VMP1_2_3RP	AGTGCCCTCTTCATCTGGAC	
PITPNC1	VMP1	VMP1_2_3FP	CTTCTGTTGGGCTTGGAACA	Control assay
PLCG1	EYA2	PLCG1_5FP	GGCACCGTCATGACTTTGTT	
PLCG1	EYA2	EYA2_3RP	CTTCCATCATAAGGCCAATGC	
PLCG1	EYA2	EYA2_3FP	TTCGTGTGGGACTTGGATGA	Control assay
HNRNPA2B1	IGF1R	HNRNPA2B1_5FP	GGAAACTTTGGTGGTAGCAGG	
HNRNPA2B1	IGF1R	IGF1R_3RP	TAAACGGCGTACTGAGTCCA	
HNRNPA2B1	IGF1R	IGF1R_3FP	GACGTCCTGCATTTCACCTC	Control assay
MSN	MCM7	MSN_5FP	CGCAAGCCTGATACCATTGA	
MSN	MCM7	MCM7_3RP	CCGGATTTTCATGCCTCGAG	
MSN	MCM7	MCM7_3FP	ATCACGGTGCTGGTAGAAGG	Control assay
STARD3	CASC3	STARD3_5FP	GCTCTGAGGCGCTACTGA	1) Same primer
STARD3	CASC3	CASC3_3RP	TTGCCTCTCTCCAGTCACAG	
STARD3	CASC3	CASC3_3FP	CAAGAGTGCTGAGGAGTCGG	2) Same primer. Control assay
STARD3	MED24	STARD3_5FP	GCTCTGAGGCGCTACTGA	1) Same primer
STARD3	MED24	MED24_3RP	ACACAGGTCCCGAGAAAAGT	
STARD3	MED24	MED24_3FP	CGTTACTAGAGCAGGCCATG	Control assay
WIPF2	ERBB2	WIPF2_5FP	CTCCTCCACCTCCCACATTT	
WIPF2	ERBB2	ERBB2_3RP	AGGTGAGTTCCAGGTTTCCC	
WIPF2	ERBB2	ERBB2_3FP	CTCCTCCTCGCCCTCTTG	Control assay
GRB7	CASC3	GRB7_5FP	GTCCTCTCTTTGTGCCACCT	
GRB7	CASC3	CASC3_3RP	TTGCCTCTCTCCAGTCACAG	
GRB7	CASC3	CASC3_3FP	CAAGAGTGCTGAGGAGTCGG	2) Same primer
ITPR1	PDHX	ITPR1_5FP	TTTCGTTTAACCTGGCCGTC	
ITPR1	PDHX	PDHX_3RP	AGGAGACAGTGATGGCATTAGT	
ITPR1	PDHX	PDHX_3FP	AGTGAGAAGGCCGTCAAGAT	Control assay
MGP	MLPH	MGP_5FP	AGCCTGATCCTTCTTGCCAT	
MGP	MLPH	MLPH_3RP	AGAGAAAGGAGGCGACGAAG	
MGP	MLPH	MGP_5RP	CTGCTGAGGGGATATGAAGGT	Control assay
NA	NA	MRPL19_F	TCGAAGGACAAGGTGTCGAG	
NA	NA	MRPL19_R	ATTCAGGAAGGGCATCTCGT	

Precursor	Mature miRNA	Primer name	Sequence
mir-21	miR-21-5p	miR21_5pF	GCAGTAGCTTATCAGACTGATG
mir-21	miR-21-5p	miR21_5pR	GGTCCAGTTTTTTTTTTTTTTTCAAC
mir-3616	miR-3616-3p	miR3616_3pF	GCGAGGGCATTTCATGATG
mir-3616	miR-3616-3p	miR3616_3pR	TCCAGTTTTTTTTTTTTTTTGCCT
mir-4714	miR-4714-3p	miR4714_3pF	CCAACCTAGGTGGTCAGAG
mir-4714	miR-4714-3p	miR4714_3pR	GGTCCAGTTTTTTTTTTTTTTTCAAC
mir-4728	miR-4728-3p	miR4728_3pF	GATCCATGCTGACCTCCCTC
mir-4728	miR-4728-3p	miR4728_3pR	GTCCAGTTTTTTTTTTTTTTTCTGGGGC
mir-106b	miR-106b-5p	miR106b_5pF	GCAGTAAAGTGCTGACAGTG
mir-106b	miR-106b-5p	miR106b_5pR	GGTCCAGTTTTTTTTTTTTTTTATCTG

Precursor	Mature miRNA	Primer name	Sequence
mir-6866	miR-6866-5p	miR6866_5pR	GGTCCAGTTTTTTTTTTTTTTTAGAATC
mir-6884	miR-6884-5p	miR6884_5pF	GAGGCTGAGAAGGTGATG
mir-6884	miR-6884-5p	miR6884_5pR	GGTCCAGTTTTTTTTTTTTTTTCAAC
mir-1343	miR-1343-3p	miR1343_3pF	GGGCCCGCACTCT
mir-1343	miR-1343-3p	miR1343_3pR	TCCAGTTTTTTTTTTTTTTTGCGA
mir-6811	miR-6811-5p	miR6811_5pF	AGATGCAGGCCTGTGT
mir-6811	miR-6811-5p	miR6811_5pR	CCAGTTTTTTTTTTTTTTTAGTGCTGT
NA	NA	SNORD48_F	GTGATGATGACCCCAGGTAACTC
NA	NA	SNORD48_R	CAGGTCCAGTTTTTTTTTTTTTTTGGTCAGA

Supplementary Table S8. Validation of fusion transcripts using real-time quantitative RT-PCR. For each fusion an assay was designed with a forward primer in the 5' partner and a reverse primer in the 3' partner. Matched positive control assays were created where one of the primers was matched with another primer in the same gene (3' partner when possible). MRPL19 was used as a positive control for all samples. Negative controls with no reverse transcriptase (No RT) and no template (No template) were included for all assays.

		Fusion transcript			Fusion partner control			MRPL19		
MicroRNA precursor	Fusion	Sample	No RT	No template	Sample	No RT	No template	Sample	No RT	No template
mir-21	PITPNC1-VMP1	28.95	NA	NA	18.90	NA	NA	21.99	39.00	NA
mir-21	B3GNTL1-VMP1	25.11	NA	NA	20.61	NA	NA	20.91	39.00	NA
mir-106b~mir-93~mir-25	MSN-MCM7	NA	NA	NA	20.59	NA	NA	21.60	36.03	NA
mir-1343	ITPR1-PDHX	NA	NA	NA	24.72	NA	NA	20.57	36.91	NA
mir-3616	PLCG1-EYA2	28.62	NA	NA	23.87	37.65	38.09	20.95	NA	NA
mir-4714	HNRNPA2B1-IGF1R	37.62	NA	NA	14.52	NA	NA	21.01	NA	NA
mir-4728	WIPF-ERBB2	26.77	NA	NA	18.73	NA	NA	20.70	NA	NA
mir-6811	MGP-MLPH	36.73	NA	NA	13.40	NA	NA	21.28	36.01	NA
mir-6866	STARD3-CASC3	22.85	NA	NA	22.79	NA	NA	22.53	39.00	NA
mir-6866	GRB7-CASC3	23.63	NA	NA	19.10	NA	NA	21.24	NA	NA
mir-6884	STARD3-MED24	26.04	NA	NA	24.67	NA	NA	22.39	NA	NA
